# Efficacy and tolerability of rituximab in patients with pemphigus foliaceus: a single-centre retrospective cohort study

**DOI:** 10.1093/skinhd/vzag025

**Published:** 2026-04-29

**Authors:** Ali Sadeghinia, Mona Tavasoli, Ahmad Vafaeian, Seyed Naser Emadi, Zeinab Aryanian, Maryam Daneshpazhooh

**Affiliations:** Autoimmune Bullous Diseases Research Center, Razi Hospital, Tehran University of Medical Sciences, Tehran, Iran; Autoimmune Bullous Diseases Research Center, Razi Hospital, Tehran University of Medical Sciences, Tehran, Iran; Autoimmune Bullous Diseases Research Center, Razi Hospital, Tehran University of Medical Sciences, Tehran, Iran; Autoimmune Bullous Diseases Research Center, Razi Hospital, Tehran University of Medical Sciences, Tehran, Iran; Autoimmune Bullous Diseases Research Center, Razi Hospital, Tehran University of Medical Sciences, Tehran, Iran; Autoimmune Bullous Diseases Research Center, Razi Hospital, Tehran University of Medical Sciences, Tehran, Iran

## Abstract

**Background:**

Pemphigus foliaceus (PF), a rare autoimmune blistering disease, still lacks a well-established optimal treatment approach. Rituximab (RTX) is not yet approved by the US Food and Drug Administration for the treatment of PF.

**Objectives:**

To evaluate the efficacy and tolerability of RTX in treating PF.

**Methods:**

In this study, all patients diagnosed with PF and treated with RTX and referred to a tertiary hospital over a period of 7 years were included. Clinical outcomes were retrospectively evaluated using Pemphigus Disease Area Index (PDAI) scores and prednisolone dosages at a minimum of four follow-up points. Patients were divided into two groups: newly diagnosed (NDP) and previously treated patients (PTP). Comparative analyses were performed across follow-up visits and study groups.

**Results:**

A total of 96 patients were included. Significant decreases in PDAI scores and prednisolone dosages were observed for all patients (both *P* < 0.0001), with no significant differences found between the study groups during a 12-month follow-up period. The NDP group exhibited a higher rate of complete remission (*P* = 0.03) and a shorter time to complete remission (*P* = 0.006) compared with the PTP group. There were no significant variations in relapse rate and time to relapse among the study groups. The most common treatment-related adverse effects of RTX were those associated with the injection, which could be managed with supportive care.

**Conclusions:**

RTX emerges as a promising therapeutic option with minimal side effects for the treatment of PF.

## Teaser

Rituximab (RTX) is not yet Food and Drug Administration–approved for pemphigus foliaceus (PF), prompting this study to evaluate its efficacy and tolerability. In a retrospective analysis of 96 patients with PF, significant reductions in Pemphigus Disease Area Index (PDAI) scores and prednisolone doses were observed (*P* < 0.0001). Newly diagnosed patients achieved higher complete remission rates (*P* = 0.03) and shorter remission times (*P* = 0.006) compared with previously treated patients, with no significant differences in relapse rates. RTX shows promise as a safe and effective treatment for PF.

What is already known about this topic?

- Pemphigus foliaceus (PF) is a rare autoimmune blistering disease characterized by scaly, crusted and erosive lesions, and an optimal treatment approach has not yet been well established.
- Rituximab (RTX), although effective and approved for pemphigus vulgaris (PV), has not yet received US Food and Drug Administration approval for PF.

What does this study add?

- As the efficacy of RTX has been demonstrated in PV, it is conceivable that similar effects may be observed in PF, given the shared pathomechanism of these two disorders.
- However, the limited number of patients with PF included in previous studies prevents the drawing of robust conclusions; furthermore, although some studies have suggested the efficacy of RTX in PF, evidence stratified by geographical regions regarding efficacy and safety remains insufficient.
- Therefore, this matched controlled study was conducted to evaluate the efficacy and safety of RTX in the treatment of PF.

Pemphigus foliaceous (PF) is a rare autoimmune blistering condition that predominantly affects the superficial layers of the skin, leading to scaly, crusted erosive lesions.^1^ The underlying pathophysiology involves the generation of autoantibodies specifically targeting desmoglein-1 (DSG1), a crucial component in maintaining the structural integrity of the epidermis.^2^ Unlike pemphigus vulgaris (PV), mucous membranes are not generally involved in PF, even in the severe forms of the disease.^3^ The different distribution patterns might be attributed to the unique expression pattern of DSG1, in contrast to desmoglein-3 (DSG3) targeted in PV but not PF, as explained by the desmoglein (DSG) compensation theory.^4,5^ The diagnosis of PF involves clinical findings, histopathological analysis of skin biopsy specimens and the identification of autoantibodies with indirect immunofluorescence, direct immunofluorescence assays and DSG enzyme-linked immunosorbent assay (ELISA).^6^

Rituximab (RTX) is a chimeric monoclonal antibody that explicitly targets CD20 molecules, which are abundantly expressed on B cells. By depleting B-cell reservoirs, RTX effectively regulates the disease. Utilizing a combination therapy of RTX and corticosteroids can be an effective treatment approach for pemphigus, allowing for lower prednisolone doses and reducing side effects compared with using prednisolone as monotherapy.^7,8^ RTX received approval from the US Food and Drug Administration (FDA) in 2018 as a first-line treatment for adult patients with moderate-to-­severe PV. While RTX is not yet US FDA-approved for the treatment of PF, current clinical guidelines recommend its use as a first-line therapy for mild-to-severe cases of PF and PV.^9,10^ Despite the noted advantages, B-cell reduction induced by RTX heightens susceptibility to viral and bacterial infections. Moreover, the cost of RTX is a barrier, and relapse rates can reach as high as 80%, presenting ongoing challenges.^11–13^

As the efficacy of RTX has been demonstrated in PV, it is conceivable that similar effects may be observed in PF, given the shared pathomechanism between these two disorders. However, the limited number of patients with PF included in previous studies prevents drawing robust conclusions.^14,15^ Furthermore, although some studies have suggested the efficacy of RTX in PF, evidence stratified by geographical regions regarding efficacy and safety remains insufficient.^16,17^ Therefore, this matched controlled study was conducted to evaluate the efficacy and safety of RTX in the treatment of PF.

## Patients and methods

This retrospective cohort study enrolled all patients diagnosed with PF who received RTX treatment at a tertiary skin hospital from April 2016 to March 2023. The diagnosis of PF was based on clinical manifestations, histopathological verification (presence of a subcorneal cleft/acantholyis), direct immunofluorescence analysis of perilesional skin (detection of IgG and complement C3 deposition in the intercellular space) and anti-DSG1 antibody serum levels.

This study included patients with a confirmed diagnosis of PF based on clinical, histopathological and immunological criteria, who required systemic treatment and either showed an inadequate response to previous therapies or had contraindications to alternative treatments. Patients were excluded if they had PV or other autoimmune blistering disorders, had active or uncontrolled infections such as tuberculosis, hepatitis B/C or HIV, had a history of malignancy within the past 5 years, suffered from severe cardiac, renal or hepatic impairment, were pregnant or breastfeeding, or had a known hypersensitivity to RTX. All patients were informed about the study protocol, and written informed consent was obtained prior to participation. Patients’ data were confidentially encoded for the study.

The criteria for initiating RTX included moderate-to-severe disease [Pemphigus Disease Area Index (PDAI) >15], inadequate response to previous treatments and contraindications to alternative therapies. Patients were categorized into two groups: newly diagnosed patients (those who received RTX within the first 6 months of the disease; NDP group) and previously treated patients (those who received RTX due to inadequate response to previous therapeutic interventions or at the time of relapse; PTP group). Before initiating RTX treatment, patients underwent a clinical evaluation where details such as age, sex, current corticosteroid dosage, disease duration, underlying medical conditions and current medication history were documented for analysis in the study. Patients were administered RTX following two distinct protocols: the modified lymphoma protocol involving 500 mg weekly for four doses and the rheumatoid arthritis protocol consisting of 1000 mg biweekly. Complete remission (CR) was assessed based on the absence of new and/or existing lesions for a minimum of 2 months off or on minimal therapy.^18^ Relapse was defined as the appearance of three or more new lesions per month that did not heal within a week, or the expansion of established lesions in a patient who had achieved disease control.^18^

As all patients were expected to have a follow-up of at least 12 months, we analysed changes in the desired variables within this period. Therefore, to evaluate the effectiveness and safety of the treatment, patients underwent regular monitoring during at least five visits: before the injection, and at 1, 3, 6 and 12 months after the final injection. Any subsequent follow-up visits were also included in the study analysis.

At each visit, patients underwent a comprehensive examination to assess relapse status, PDAI score, corticosteroid dosages administered and any associated side effects. Side effects were assessed during and after each injection session. Anti-DSG1 antibody levels in the patient’s serum were assessed using the ELISA method (EUROIMMUN, Lübeck, Germany) during follow-up visits, if possible. As anti-DSG1 levels were not available for all follow-up timepoints, the timepoints were categorized into three distinct phases: diagnosis, CR achieved during the study and relapse occurring during the study. Furthermore, age, sex, RTX dosage at each visit, location of persistent lesions, current and previous medications, and past medical history were recorded.

Mean and SD were presented for continuous variables, while frequency and percentage were provided for discrete variables. Two-way Anova test with repeated measures has been used to analyse PDAI and prednisolone dosage changes over timepoints and between study groups, with Bonferroni adjustments applied for post hoc tests. The comparison of quantitative variables between groups utilized independent and paired *t*-tests, and Wilcoxon rank sum test, while discrete variables were evaluated using χ^2^ and Fisher’s exact tests. The time to CR and time to relapse in the two RTX regimen groups were visualized using Kaplan–Meier curves and analysed using the log-rank test. A significance level of 0.05 was set for this study. All statistical calculations were performed in the R open-source environment (R Foundation for Statistical Computing, Vienna, Austria).

## Results

### Patient demographics and baseline characteristics

In this study, 96 patients diagnosed with PF were included, comprising 53 women and 43 men. The mean (SD) age of the whole study population was 45.85 (14.85) years at the study onset. The patients were followed for a mean duration of 20.32 months. All patients were monitored for a minimum of 12 months, with 41 patients being monitored for over 12 months (month 1, month 3, month 6, month 12 and the last visit).

### Underlying medical conditions and concomitant medications

Forty-two (*n* = 42/96; 44%) patients presented with underlying medical conditions, with high blood pressure (*n* = 12; 13%), hyperlipidaemia (*n* = 11; 11%), diabetes (*n* = 9; 9%) and hypothyroidism (*n* = 9; 9%) being the most prevalent (Table 1). The most common prescribed medications were atorvastatin/rosuvastatin (*n* = 9; 9%) and levothyroxine (*n* = 8; 8%). Thirty-three patients had previously been receiving systemic adjuvant immunomodulatory medications (Table 1). The primary prescription drugs were mycophenolate mofetil and azathioprine (Table 1). Of the six patients who had been receiving methotrexate prior to the study, one continued its use during the study. Treatment complications were observed in 11 patients (11%), with steroid acne (*n* = 5; 5%), herpes zoster (*n* = 2; 2%), avascular necrosis (*n* = 2; 2%) and tinea versicolor (*n* = 2; 2%) being the most common. Sore throat (*n* = 2; 2%) and flushing (*n* = 2; 2%) were the most common side effects associated with infusion (Table 1).

**Table 1:** Medical history, including medications and treatment complications in the study population (*n* = 96)

	*n* (%)
Past medical history	
Hypertension	12 (13)
Hyperlipidaemia	11 (11)
Diabetes mellitus	9 (9)
Hypothyroidism	9 (9)
Ischaemic heart disease	2 (2)
Benign prostatic hyperplasia	2 (2)
Peptic ulcer disease	2 (2)
Drug history	
Atorvastatin/rosuvastatin	9 (9)
Levothyroxine	8 (8)
Losartan	4 (4)
Metformin/zipmet	4 (4)
Pantaprazol	2 (2)
Amlodipine	2 (2)
Previous systemic adjuvant medications	
Mycophenolate mofetil	16 (17)
Azathioprine	12 (12)
Methotrexate	6 (6)
Ciclosporin	3 (3)
Cyclophosphamide	1 (1)
Infusion-related adverse events	
Sore throat	2 (2)
Flushing	2 (2)
Chest tightness	1 (1)
Nasal obstruction	1 (1)
Urticaria	1 (1)
Headache	1 (1)
Pruritus	1 (1)
Treatment complications	
Steroid acne	5 (5)
Herpes zoster	2 (2)
Avascular necrosis	2 (2)
Tinea versicolor	2 (2)
Mucosal candidiasis	1 (1)
Cellulitis	1 (1)
Urinary tract infection	1 (1)
Abscess	1 (1)
Fatty liver	1 (1)
Elevated liver function test	1 (1)
Striae	1 (1)

Injection-related adverse events were mild and were managed with antihistamines, hydrocortisone and supportive care (Table 1). Furthermore, no other serious reactions were observed in association with the treatment process (Table 1). Six cases of infection, including cellulitis, abscess, urinary tract infection, herpes zoster, tinea versicolor and oral candidiasis, were recorded and successfully treated. Moreover, the frequency of complications was not significantly different in women compared with men (*P* = 0.95). No significant disparities were observed in the incidence of side effects between the two injection protocols (*P* = 0.77).

### Comparison of study groups at baseline

In the comparison between the two study groups, no significant differences were found in terms of age, sex, anti-DSG1 levels at diagnosis and relapse, relapse rate, cumulative prednisolone and RTX dosages over the course of study, RTX treatment protocol and the incidence of treatment complications (Table 2).

**Table 2:** Comparative analysis of study group characteristics

Variable	All patients (*n* = 96)	NDP (*n* = 56)	PTP (*n* = 40)	Statistics	95% CI	*P*-value
Age (years), mean (SD)						
	45.85 (14.85)	45.25 (16.28)	46.72 (12.66)	1.47^a^	−7.37, 4.44	0.62
Sex						
Female	53 (55)	28 (50)	25 (63)	0.60^b^	0.24, 1.48	0.31
Male	43 (45)	28 (50)	15 (38)			
Anti-DSG1 levels, mean (SD)						
Diagnosis	189.61 (43.64)	189.25 (34.76)	190.02 (52.60)	0.00^c^	0.00, 0.00	0.88
Complete remission	47.49 (73.19)	22.75 (48.19)	82.42 (88.53)	−19.32^c^	−161, −3.50	0.005**
Relapse	151.91 (74.97)	156.67 (75.06)	150.12 (80.04)	0.00^c^	−130.00, 155.00	0.91
RTX protocol						
Modified lymphoma	68 (71)	40 (71)	28 (70)	1.07^b^	0.40, 2.85	>0.99
Rheumatoid arthritis	28 (29)	16 (29)	12 (30)			
Cumulative corticosteroid dose (g), mean (SD)						
	3.27 (3.25)	2.73 (2.01)	4.00 (4.34)	−0.37^c^	−1.43, 0.38	0.36
Cumulative RTX dose (g), mean (SD)						
	2.53 (1.16)	2.33 (0.76)	2.80 (1.52)	0.00^c^	0.00, 0.00	0.32
Complete remission						
Yes	80 (83)	51 (91)	29 (73)	0.26^b^	0.06, 0.92	0.03*
Relapse						
Yes	25 (26)	11 (20)	14 (35)	2.20^b^	0.79, 6.20	0.15
Persistent lesions						
Arm	1 (7)	0 (0)	1 (13)			0.04*
Face	2 (13)	2 (29)	0 (0)			
Nose	2 (13)	2 (29)	0 (0)			
Scalp	10 (67)	3 (43)	7 (88)			
Treatment complications						
Yes	17 (18)	10 (18)	7 (18)	0.98^b^	0.28, 3.19	>0.99

CI, confidence interval; DSG1, desmoglein-1; NTP, newly treated patients; PTP, previously treated patients; RTX, rituximab. ^a^Difference in mean; ^b^odds ratio; ^c^location shift. *P < 0.05, **P < 0.01.

### Anti-desmoglein-1 levels over time

There was no statistically significant difference in anti-DSG1 levels between the two study groups at the time of diagnosis (*P* = 0.88) and relapse (*P* = 0.91). However, at the time of CR, mean (SD) anti-DSG1 levels were significantly higher in the PTP group than in the NDP group [82.42 (88.53) vs. 22.75 (48.19), *P* = 0.005] (Table 2).

### Clinical response and relapse rates

**Figure 1:**
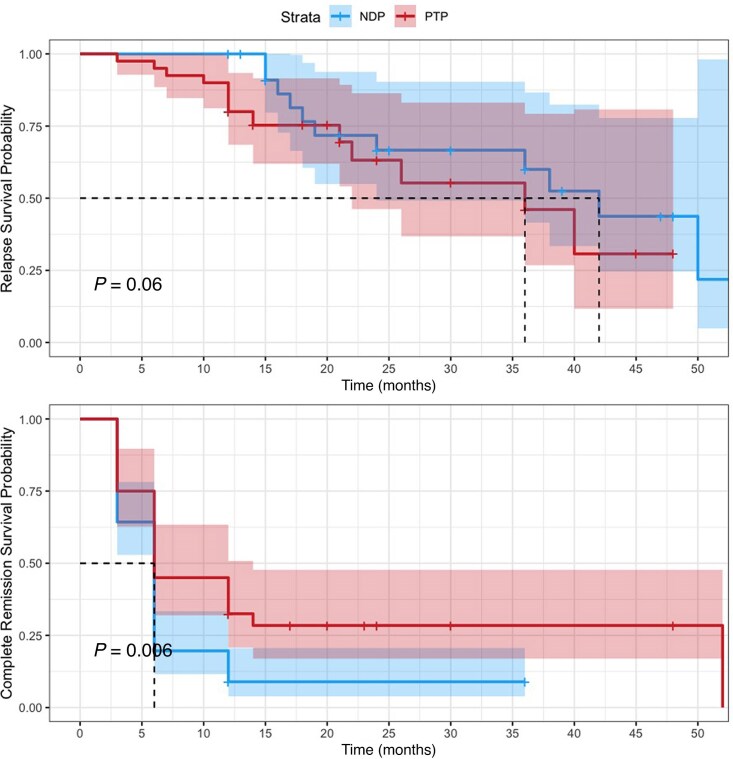
Kaplan–Meier curves showing the comparative analysis of time to relapse and time to complete remission between two rituximab indications. NDP, newly diagnosed patients; PTP, previously treated patients.

Eighty (83%) patients attained CR. The frequency of CR was significantly lower in the PTP group compared with the NDP group (odds ratio 0.26, *P* = 0.03) (Table 2). Additionally, the mean (SD) time required to achieve CR was significantly shorter in the NDP group compared with the PTP group [5.53 (2.77) vs. 7.86 (9.16) months, *P* = 0.006] (Figure 1). Twenty-six per cent (*n* = 25) of the patient cohort encountered relapses, with a mean time to relapse of 20.92 (12.55) months post-RTX treatment. The relapse rate (*P* = 0.15) and time to relapse (*P* = 0.06) were not significantly different between the study groups (Table 2, Figure 1).

### Pemphigus Disease Area Index scores and prednisolone dosage trends

**Figure 2:**
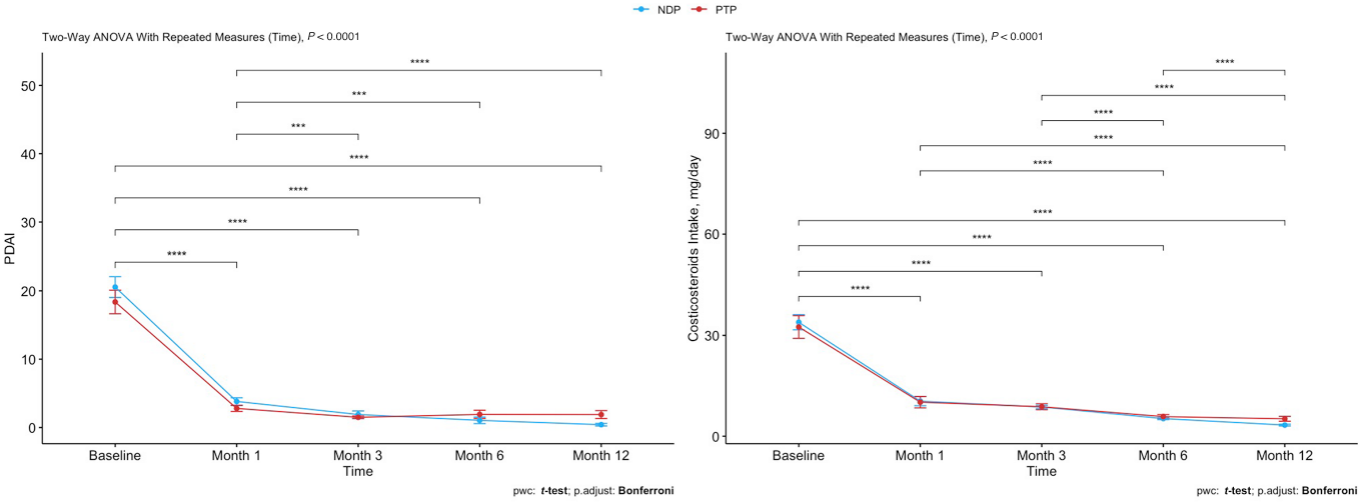
Comparative analysis of Pemphigus Disease Area Index (PDAI) scores and daily corticosteroid dosages across rituximab indications and various timepoints. NDP, newly diagnosed patients; PTP, previously treated patients; pwc, pairwise comparisons. ***P < 0.001; ****P < 0.0001.

The PDAI score demonstrated a significant decline across all patients from treatment initiation to the 12-month assessment visit (*P* < 0.001) (Table 3, Figure 2). Notably, no significant difference in PDAI levels was observed between the two study groups, either overall (*P* = 0.78) or at individual timepoints (*P* = 0.22) (Table 3). Additionally, there was a significant decrease in prednisolone dosage from the beginning of treatment to the 12-month follow-up visit for all patients (*P* < 0.001) (Table 3, Figure 2). Despite this trend, there were no significant changes in prednisolone dosage between the NDP and PTP groups, either overall (*P* = 0.86) or at individual timepoints (Table 3, Figure 2).

**Table 3:** Pemphigus Disease Area Index (PDAI) and prednisolone dosage in the study groups at different timepoints

	Baseline	Month 1	Month 3	Month 6	Month 12	*P*
PDAI						
All	19.65 (11.18)	3.40 (3.60)	1.74 (3.08)	1.42 (3.68)	1.04 (2.68)	Group: 0.78
NDP	20.55 (11.40)	3.82 (4.05)	1.91 (3.89)	1.05 (3.54)	0.42 (1.36)	Time: <0.001
PTP	18.38 (10.89)	2.80 (2.78)	1.50 (1.26)	1.93 (3.87)	1.90 (3.67)	Interaction: 0.22
Prednisolone dosage (mg daily)						
All	33.32 (18.83)	15.46 (8.65)	8.72 (4.52)	5.49 (2.97)	4.09 (3.41)	Group: 0.86
NDP	33.91 (16.95)	16.29 (8.04)	8.68 (3.59)	5.25 (2.30)	3.30 (1.90)	Time: <0.001
PTP	32.50 (21.39)	14.28 (9.42)	8.76 (5.62)	5.83 (3.71)	5.18 (4.58)	Interaction: 0.66

Data are presented as mean (SD). NTP, newly treated patients; PTP, previously treated patients.

## Discussion

The adverse effects associated with corticosteroids have prompted a shift in the therapeutic approach for patients with pemphigus, transitioning from sole corticosteroid treatment to combination regimens incorporating immunosuppressive agents.^9^ Nonetheless, immunosuppressive therapies have limitations, resulting in high relapse rates and adverse events.^19–25^ This retrospective cohort study was conducted to evaluate the efficacy and adverse effects of the RTX in patients diagnosed with PF. Additionally, it compared the outcomes between patients who were newly diagnosed and received RTX as initial treatment and those who underwent RTX therapy following unsuccessful prior treatments. Assessment of efficacy included monitoring PDAI scores, anti-DSG1 levels and corticosteroid dosages.^26,27^

The application of RTX, a monoclonal antibody specifically directed at CD20 on B lymphocytes, represents a groundbreaking advancement in the management of bullous autoimmune disorders, particularly in the context of PV and, subsequently, PF.^28,29^ Previous research has underscored the efficacy of RTX and its importance in facilitating CR in a substantial portion of patients, particularly those with treatment-resistant conditions. This results in sustained clinical remission over an extended period, decreased reliance on prednisolone and minimized adverse effects.^28,30^ Given the favourable outcomes observed with RTX in PV, our investigation delved into the application of RTX in PF, a variant that has received comparatively limited attention in the RTX therapy.

The study demonstrated a significant reduction in PDAI scores and prednisolone dosage in patients with PF following the administration of RTX. We found a CR rate of 83% during a 12-month follow-up in patients with PF who received RTX. Comparably, a study demonstrated that 50% of 12 patients with PF achieved CR following their initial RTX infusion.^31^ In a systematic review involving 105 patients with PF, 63% of the 76 patients treated with RTX achieved CR over a 22-month follow-up period.^16^ Moreover, a study involving 108 patients with pemphigus (92 PV, 16 PF) reported an overall remission rate of 74%.^32^ Other studies have reported a CR rate of approximately 90% for PV at a 2-year follow-up.^11,33^ The incidence of CR after RTX injection in patients with PF may be comparatively lower, consistent with our report.^31,34^ In accordance with the existing literature,^32,35,36^ our study revealed a higher probability of achieving CR in the NDP group compared with the PTP group. Notably, prior research suggests that previously treated patients may exhibit a more refractory disease state and may be inherently poorer responders,^37^ which could explain the longer time to CR observed in this group. Our findings and prior evidence collectively advocate for the superior outcomes associated with using RTX as an initial treatment. However, we observed that the time to relapse and the rate of relapse were not significantly different between the two groups (Figure 1). Aside from statistical power, this could be due to the cumulative immunosuppressive effect of prior RTX cycles in the PTP group, which may have delayed relapse and lowered the relapse rate despite the more severe form of the disease, making them comparable to the NDP group.

The mean time to CR among those who attained it was 6.38 months, which aligns with findings from other studies that have reported comparable times for achieving CR, ranging from 3 to 9 months.^30,31^ However, prolonged time to CR has also been documented mainly in the context of PTP.^34^ We observed a longer time to CR in the PTP group. Furthermore, we observed a relapse incidence of 26% (*n* = 25/96), whereas a rate of 39% has been reported with longer follow-up durations.^16^ In a recent study, patients with PF receiving RTX showed a lower relapse rate and a higher rate of sustained CR compared with those with PV, while most of the observed serious adverse events were associated with corticosteroids.^14^ Given the high probability of relapse within 24 months, the rationale for RTX use as maintenance therapy between 18 and 24 months becomes increasingly compelling. The longer time to CR among patients in the PTP group underscores the necessity of maintenance treatment, especially for this subgroup, albeit at a later stage than in PV.

The side effects attributed to RTX have emerged as a significant focal point for medical practitioners.^29^ However, a significant proportion of these adverse events is related to the infusion procedure and can be efficiently managed through established protocols, including modifying the infusion rate and employing supportive measures such as chlorpheniramine, hydrocortisone and acetaminophen as premedication.^38^ Consequently, the key focus is on effectively managing these side effects while recognizing that they do not undermine the effectiveness of the treatment.^38–40^ The most common treatment-related adverse effects observed in our study, including steroid acne, herpes zoster, avascular necrosis and tinea versicolor, could be attributed to current or prior corticosteroid use (Table 1). Although the majority of infusion-related adverse effects in our study were mild, our findings underscore the importance of careful patient monitoring and the implementation of effective management strategies.^41^

Limitations of our study include its retrospective nature, which may have introduced selection bias. Only patients with complete medical records were included. This selection criterion might have favoured patients with more difficult-to-manage pemphigus who required frequent follow-ups and monitoring. Lack of a comparative arm and single-centre context are other limitations. Conversely, its strengths comprise a sizable cohort and extensive follow-up period. Considering the chronic nature of autoimmune disorders, including pemphigus, long-term effects deserve attention, particularly regarding the duration of remission and the safety of treatment, which are essential areas for future investigation.

Our study demonstrates favourable efficacy and safety outcomes with RTX use in patients with PF, consistent with findings from other recent studies.^14,15^ Notably, patients who are newly diagnosed and receiving this drug exhibit higher CR rates and shorter time to achieve CR. Based on the present findings and the potential benefits, including the high overall success rate and the opportunity it provides to cautiously reduce corticosteroid dosages, RTX may be considered a promising first-line treatment option for patients with PF. Nevertheless, the optimal infusion timing and patient cohorts most suited for reinfusion remain areas requiring further clarity, necessitating expansive, prolonged studies to resolve these uncertainties.

## Data Availability

The data underlying this article will be shared on reasonable request to the corresponding author.
